# When Models Interact with Their Subjects: The Dynamics of Model Aware Systems

**DOI:** 10.1371/journal.pone.0020721

**Published:** 2011-06-15

**Authors:** Dervis Can Vural

**Affiliations:** Department of Physics, University of Illinois at Urbana-Champaign, Champaign, Illinois, United States of America; Universita' del Piemonte Orientale, Italy

## Abstract

A scientific model need not be a passive and static descriptor of its subject. If the subject is affected by the model, the model must be updated to explain its affected subject. In this study, two models regarding the dynamics of model aware systems are presented. The first explores the behavior of “prediction seeking” (PSP) and “prediction avoiding” (PSP) populations under the influence of a model that describes them. The second explores the publishing behavior of a group of experimentalists coupled to a model by means of confirmation bias. It is found that model aware systems can exhibit convergent random or oscillatory behavior and display universal 1/f noise. A numerical simulation of the physical experimentalists is compared with actual publications of neutron life time and 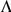 mass measurements and is in good quantitative agreement.

## Introduction

The notion that the act of observation can alter the observed system is familiar to any contemporary scientist. Less familiar is the notion that a model can alter the system it aims to describe. Such model-system couplings may be substantial for cognitive [1], [2], social [3]–[7], or economic [8]–[10] systems in which the constituent agents have full or partial access to the model that represents them. It is conceivable for example, that an analysis of word frequencies in spoken or written language [11] can alter the behavior of those who produce these words; an analysis of stock market [12] can alter the behavior of stock traders; and an analysis of fashion [13] can alter the behavior of those who influence fashion. The “physical sciences” do not seem to be free of model-subject interaction either, since experimenters are influenced by theory through confirmation bias [15] and theorists build new models based on these experiments.

The interaction between subjects and their models has long been recognized in separate disciplines, and referred as “the looping effect” [7] in philosophy, “the enlightenment effect” [3] in psychology, and “performativity” [8] or “virtualism” [9] in economics. The model aware behavior in the physical sciences has been recognized too, and rightfully renounced as “confirmation bias” [14], [15]. Unfortunately, the cited considerations are very qualitative, and discipline-specific. Presently there exists no quantitative and generally applicable theory of model awareness. Without such a theory, it is not possible to ask whether the description of a model aware system will converge to a fixed point, or perpetually change while manipulating its subject. Neither is it possible to ask how the behavior of a system changes with increasing model awareness, or what universal properties, if any, do model aware systems have.

The present study aims to answer these questions, at least partially, by introducing two quantitative models of model awareness, cast as much as possible in a discipline-independent language. The first describes a population of prediction seeking or prediction avoiding agents who update their behavior at every time step depending on the current model that describes them. The second describes the behavior of a population of experimental physical scientists, who decide whether to publish or not depending on the proximity of their data to the current “model”. In both the physical and social case, populations provide feedback to the models too, since models must be updated to explain the current state of the population. The outcomes are then compared against two behavioral experiments [1], [2] qualitatively, and particle physics data [16] quantitatively.

Even though real-life models usually involve elaborate verbal descriptions, and/or sophisticated mathematical machinery, in this study we will consider much simpler ones such as propositions regarding majority behavior, or averages of multiple publications regarding a physical quantity. As simple as they may be, it seems appropriate to refer to them as “models” since they are falsifiable descriptions with predictive power.

## Analysis

### Model Aware Social Populations

There has been a number of agent-based herding/anti-herding approaches in the literature used to explain a wide variety of social and economical phenomena (the interested reader is referred to [17]). The present approach is reminiscent of a Polya urn process [18], in the sense that we will be modifying an ensemble according to its sampled outcomes.

We consider a population of 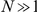, in which an individual can be in either one of the states 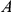 or 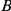. These states can represent any opinion, property and behavior. At any given time 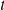 the state of the population can be characterized by the fraction of 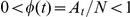 of individuals in state 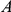. After each time step, a scientist performs a measurement by choosing 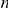 random individuals (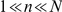) out of 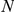 and publishes whether the majority of 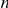 is 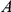 or 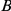. In connection with our introductory remarks we take any statement regarding majority behavior as a model; one example may be “investors avoid risk”.

When the model is published, each individual among 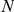 may become “aware” of the model with probability 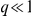, and subsequently update his or her state. The state of those unaware of the model is assumed to remain unchanged.

Two types of populations will be considered separately, defined in terms of how the aware individuals update their state: A *prediction seeking population* (PSP) is one in which the aware individuals align their state with the prediction of the published model. For example, if the publication reports that “most people are 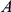
”, then the aware 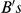 become 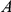 in the next step (the aware 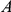
's remain unchanged). A *prediction avoiding population* (PAP) is one in which individuals anti-align their state. For example, if the publication reports that “most people are 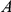
”, then the aware 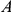
's flip their state to 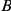 (the aware 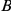
's remain unchanged).

Once the aware subpopulation updates its state, a subsequent scientist will be sampling, measuring and modeling a population of different nature. We will consider the Markovian dynamics of 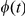 over many such iterations. Unlike a Polya process, our ensemble is fixed in size, the number of modified agents is nondeterministic, and the modification is done according to a majority rule.

Let us first focus on a PSP. Given the state 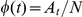, the probability of having 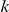 people who are A's in a sample of 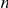 is given by the hypergeometric distribution,



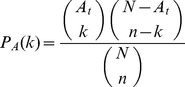
(1)Without loss of generality, suppose 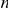 is odd. The probability that the majority of the scientist's measurement sample is 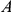 is



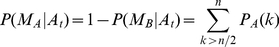
(2)After the publication, the probability 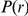 that 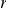 people will become aware of the result is



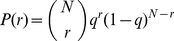
(3)Given that there exists 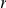 aware individuals, and given the majority of the sample is 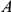, the probability that 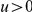 of them (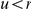) to change into 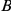 is



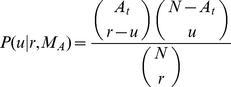
(4)Thus, the probability that the population changes from 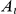 to 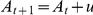 is given by the transition matrix



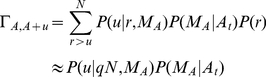
(5)Similarly, the probability that 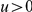 individuals change in the other direction is



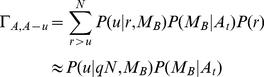
(6)where this time,



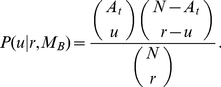
(7)We immediately see that if 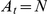, 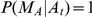, 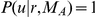 and 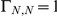. The same holds true if 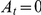, and 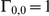. Thus, 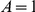 and 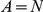 are the absorbing states of the corresponding Markov Chain.

In the case of PAP, regardless of what the majority in the selected sample is, there always is a nonzero probability that the aware population will include some of individuals from the majority state, who in the next time step will flip. Any accidental trend due to finite sampling 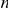 is counter balanced in the next time step by the prediction avoiders, who consequently cause an anti-trend. Thus, a PAP state will fluctuate on average by 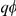 (though 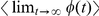 exists; cf. below). The maximum expected change 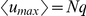 will occur when 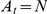 or 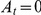.

**Figure 1:**
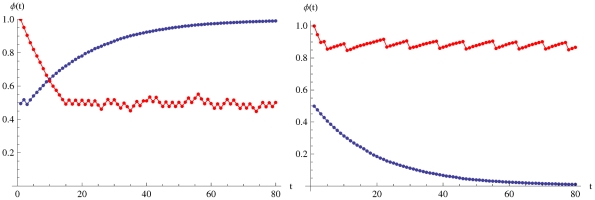
Fraction of individuals 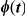 having a certain quality 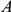 as a function of time 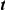. Prediction seeking (blue) and prediction avoiding populations (red) evolve under the influence of an non-biased model (left) and a biassed e = 0.45 model (right). Here, 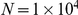, 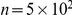, 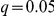.

Let us now consider the expectation value 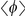. For a PSP the expectation value of the change 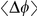 is 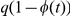 if 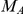, and 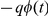 if 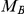. Therefore,



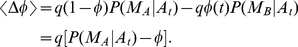
(8)Similarly, for a PAP 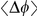 is 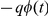 if 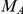 and 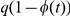 if 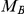. Therefore,



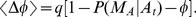
(9)The function 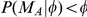 for 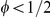, 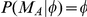 for 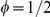 and 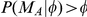 for 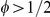. Thus, the 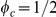 is a first order equilibrium state for both PSP and PAP. For a PSP, 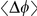 is positive for 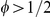 and 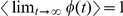 if 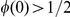. 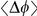 is negative for 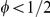, and 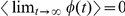 if 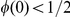. In contrast, for a PAP, 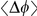 is negative for 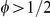 and positive for 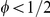; hence 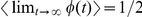 regardless of the initial state. Note that on average, the variance 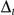 within the population is monotonically decreasing for a PSP, and monotonically increasing for a PAP (Fig (1)).

It is also interesting to study the effects of incorrect models. This could happen for example due to an asymmetry in identifying one of the states, or misinterpreting correctly measured data during “modeling”. Suppose that the scientist publishes 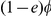 instead of 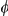, the effect of which can be taken into account by modifying 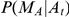;



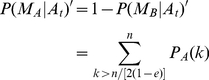
(10)


**Figure 2:**
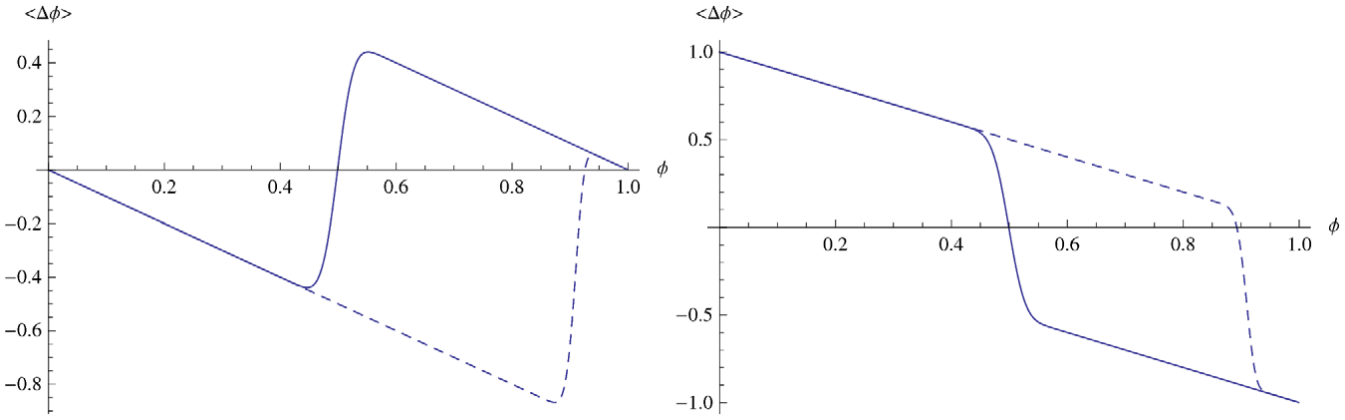
〈
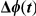
〉 as a function of 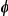. Prediction seeking (left) and prediction avoiding populations (right) in the presence of error 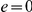 (solid) and 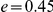 (dashed). 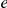 shifts the equilibrium point for both PSP and PAP, and causes regular oscillations in PAP. Plot parameters are 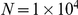, 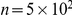, 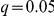.

By observing 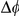 vs 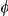 (cf. eqn(8),(9) and Fig (2)), we see that 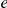 shifts the equilibrium point 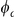 for both PAP and PSP. As a result, the PSP may now cross 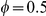, and unlike the 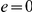 case the variation within a PSP population need not monotonically decrease. The absorbing states of a PSP does not change, whereas for a PAP 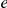 simply shifts the value of 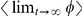.

**Figure 3:**
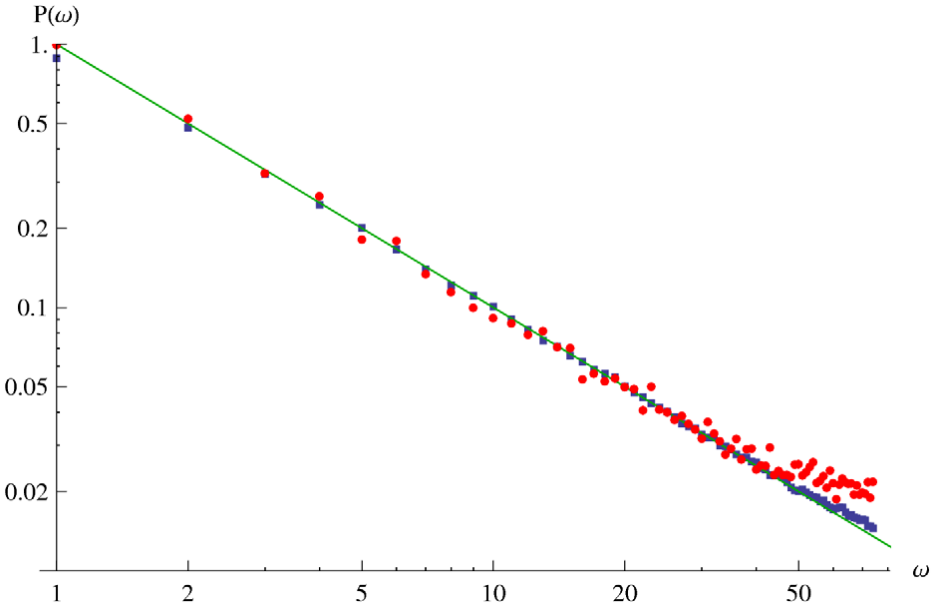
The power spectrum 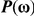 of prediction avoiding (red) and prediction seeking (blue) populations. It can be seen that both populations obey 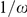 statistics (green). Here 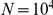, 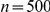 and 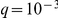.

Numerical simulations are carried out for 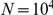, 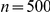, 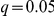, and reveal interesting features regarding fluctuations (Fig (1), Fig (3)): In a PAP, as the error 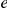 is gradually increased within 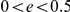, the period of the small fluctuations in equilibrium dramatically increase and regularize (Fig (1)). The reason is evident in Fig (2); for 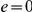, we have 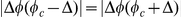, and therefore the period of trend/anti-trend fluctuations is equal to one time step. On the other hand for 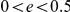, the difference in 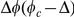 and 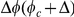 leads to slower rises following rapid drops. As we continue to increase 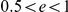, eqn(9) now has a stable point, and the PAP starts to display convergent behavior much like the PSP. Thus, one could say that an unchanging model of a PAP is only possible if the model is practically wrong.

A more interesting feature is that despite their opposite nature, PSP's and PAP's both appear to exhibit a 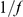 noise in their power spectrum 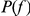 if 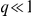 (Fig (3)). The 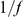 noise is the signature of self-organized critical systems [19] and is ubiquitous in nature (see for eg. [20]–[22]). We predict that the same should appear in measurements of social model aware populations.

### The Model Aware Behavior of Physical Scientists

Ideally, separate measurements of a physical quantity typically fluctuates around a fixed value according to a normal distribution due to random measurement errors. However, since experimenters tend to be influenced by past measurements and established theoretical models, data points are highly correlated [15] and have visible systematic trends over time [16]. This may be because, if the outcome of an experiment is significantly different from an accepted model or past outcomes, the experimenters may be “improving” their setup by eliminating some of the systematic errors which otherwise average out to zero, or even by simply repeating the experiment. In turn, future models are built on experimental data that have already been influenced by past models. Thus, it seems that the presence of model aware behavior extends beyond social sciences.

We describe the behavior of a population of model aware physical scientists with the following simplified characteristics: An experimenter measures a physical quantity 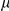 at time 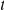 and obtains 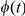 in order to publish the 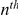 paper 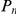 on the subject. Because of the precision limitations of the apparatus, 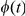 is assumed to be normally distributed around 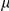 with standard deviation 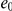. After taking the measurement, the experimenter compares the outcome with the average 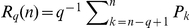 of 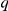 preceeding published measurements, with standard deviation 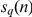.

In this case we define the “model” to be 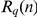 (or any set of verbal or mathematical axioms that reproduce it). If 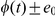 does not overlap with 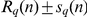, the experimentalist suspects there might be a mistake in the setup and decides to repeat the experiment at 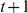. If on the other hand, if 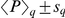 overlaps with the “model”, he publishes 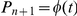. It is assumed that 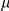 is independent of time, and that non-random errors are negligible.

Since, the random variable 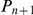 depends on the past 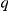 publications 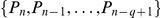 and is distributed according to a “partial Gaussian” function, we can write



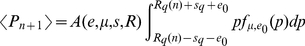
(11)where 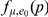 is the usual Gaussian distribution with mean 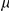 and standard deviation 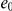. Since the possible publications are limited by the scientist's confirmation bias, the normalization constant 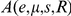 is determined by 
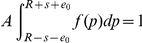
.

We can immediately see that when 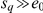, the lhs of (11) is precisely 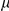 and 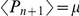 regardless of 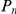; in other words, a lack of scientific consensus speeds up model convergence! The integral and 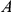 can be calculated exactly,



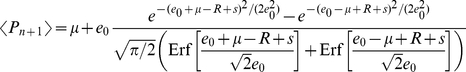



**Figure 4:**
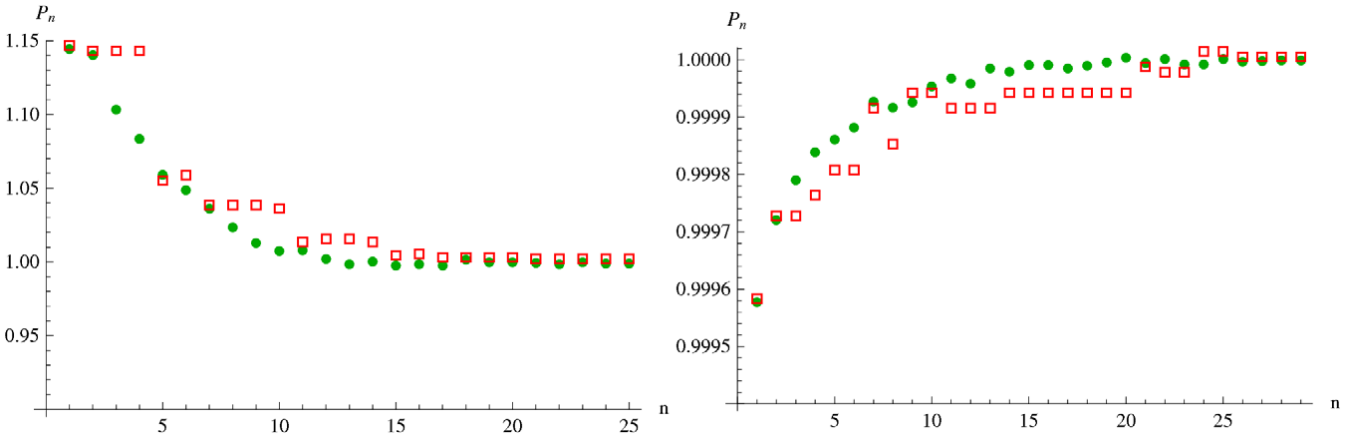
Published values of a physical quantity 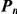 at publication number 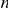 **.** Here, the Average values of publications of a simulated population of physical scientists (circles) is compared with actual [16] particle physics measurements (boxes) of neutron life-time (left) and 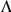 mass (right) normalized by their last value. 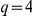 is chosen for the neutron measurements and 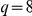 for the 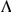 measurements. The two initial points are taken from data and used as initial conditions in the simulation.

Note that if 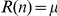 we get 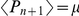, and then 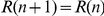. Also, note that if 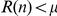 we have 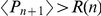, and as a result 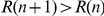. On the other hand if 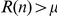 we have 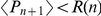 and as a result 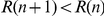. Thus 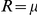 is a stable equilibrium point. Furthermore, the quantity 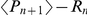 is linear in 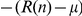 up to third order in 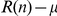, hence for values 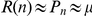 we have exponential convergence (cf. Fig (4)).

**Figure 5:**
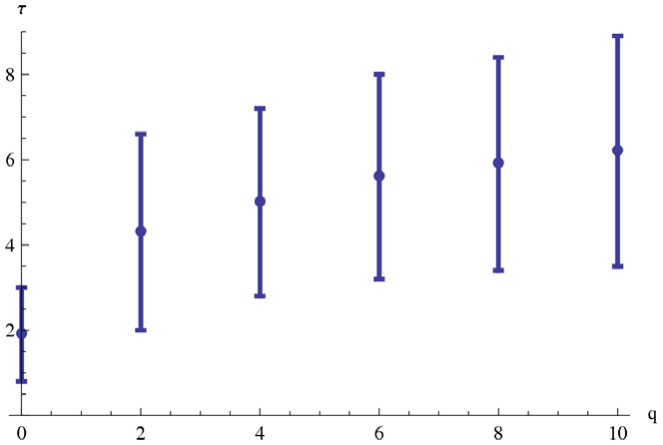
Average convergence time 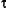 as a function of model awareness 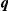. Here two identical measurements with 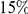 error is taken as initial conditions. The error bars indicate the standard deviation in 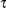.

Fig (4) shows the outcomes of exact numerical simulations and compares them with historical publications. The first two data points are taken from actual particle physics experiments [16] as an input, and the rest of the data points are iterated. The short time behavior is characterized by tight clusters separated by abrupt jumps. The long time behavior approximately fits an exponential relaxation 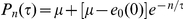 curve. The convergence time 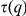 determined from a least square fit, is a random variable with mean and standard deviation plotted as a function of 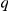 (Fig (5)). The convergence times increase and diversify with increasing model awareness of the experimenters. We conclude that model aware experimenters considerably slow down scientific progress.

## Discussion

To further connect model awareness to the experimental literature, two recent behavioral studies [1], [2] will be discussed within the PSP/PAP framework and will be compared with the simulation results. Due to lack of time dependent behavioral data, this comparison will have to be qualitative. Fortunately it will be possible to compare the outcomes of the simulations of the physical scientists quantitatively, since there exists records of historical particle physics publications [16].

The first behavioral study is one on the behavioral effects of *belief* of testosterone administration and actual testosterone administration [2]. It was found that women who *believed* that they were given the hormone behaved significantly more “unfair” than those who *believed* they were not given the hormone (regardless of whether or not they actually were). Curiously, the effect of actual testosterone was to increase fairness.

To measure the effect of belief of testosterone administration, is essentially to measure the effect of the *model* of testosterone on its model aware subject. The model of testosterone is the (as it turns out, incorrect) statement that “testosterone causes people to be unfair”. The subjects of this study were familiar with this model, and behaved in accordance with its prediction. However, the study not only measures the effect of the testosterone model, it also alters it (i.e. by publicizing that testosterone actually increases fairness). Therefore if one repeats the same experiment in a few years, one may find that the effect of the model is exactly opposite, since if 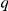 is large enough some participants may be familiar to the altered model of testosterone.

The second behavioral study is one on the effect of “sophistication” (defined as having knowledge on psychology and elementary statistics) on the ability of generating random numbers [1]. Here it was found that sophistication can dramatically reduce -if not entirely eliminate- the many nonrandom trends non-sophisticated people tend to display. Some such trends include avoiding repetitive triplets (such as 8,8,8) or favoring descending sequences (such as 5,4,3) over ascending ones (such as 3,4,5). Here too, what is measured is the influence of a model, which in this case, describes how people generate random numbers. The people labeled “sophisticated” are aware of this model, and actively avoid its predictions. Furthermore, if 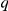 is large enough, upon repeating the experiment at a later time one might observe “super-sophisticated” subjects: Those who are aware of and avoid the model predicting a population of a mixture of non-sophisticated and sophisticated individuals.

Both experiments have two data points that are useful for us. The belief of testosterone administration activates the present stage of a long model aware evolution in a PSP. Similarly the “sophistication” variable in the random number study is a marker of a similar final state of a PAP. Thus one may qualitatively compare 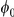 to 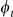 and 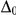 to 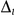, where 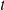 is the present time. The short term behavior of the simulations qualitatively agree with both experiments [1], [2], the common features of which are (i) the model aware subjects are significantly different than the non model aware subjects and (ii) the non model aware subjects are more similar among themselves than model aware subjects.

It is appropriate to represent the model aware subjects of the testosterone study (i.e. those who believe they received testosterone) by a PSP evolving under a strongly biased model. The simulation agrees with feature (i) and for short times, feature (ii). For long times, the simulated PSP eventually becomes less diverse than its starting state. Perhaps continued publications of studies similar to [2] over the years, will reveal this time dependence. It is appropriate to represent the model aware subjects of the random number study [1] with a PAP evolving under an unbiased model. Here too, the simulation agrees with feature (i) and feature (ii) (remember that 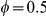 represents a maximally diverse population).

The actual neutron half-life and 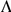-mass measurements from 1960 to 2010 as reported in [16] are compared with the outcomes of the simulations, and is good agreement (Fig (4)). It is very interesting that other historical particle physics data reported in [16] fits reasonably well to the same exponential form. The average convergence time 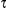 depends on 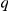. For example, since 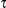 is larger for 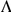 mass measurements compared to neutron life-time measurements, one can conclude that the scientists measuring 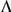 mass were more model aware.

### Conclusion

It was shown that for physical scientists and prediction seeking populations, models and their subjects can co-evolve to a consistent state. Loosely speaking, for these systems model awareness sets a lower time limit to reach “truth”. For prediction avoiding populations, such a consistent state is possible only if the systematic error in the model is large enough (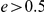) to make the model practically incorrect. Thus, a PAP, despite evolving according to deterministic laws, is either unpredictable, or falsely predictable due to the subject-model interaction.

The 1/f spectrum observed in populations of opposite nature suggest that there may be other universalities common to all model aware systems.

While this is just a preliminary study, the simulations presented here demonstrate that the models and their subjects can be highly coupled and can radically alter each other. Since the interaction of subjects with models seem to be present in a very diverse range of fields, the framework proposed was intentionally kept simple. This way, the theorists of different fields can add relevant system-specific details, and study the variants of the proposed model. Such variants could include interaction between individuals, noise, or variable model errors.

Our study motivates a wide range of additional questions that can be explored theoretically and experimentally: What is the influence of a time dependent model? Is it possible to construct a model that takes into account its own influence? Can the above considerations be generalized to more realistic models involving not mere numbers, but simulations or equations? How about model aware systems such as the stock market, where the subject is under the influence of multiple models? Hopefully the present work will inspire the scientific community for a deeper exploration of model aware systems.
